# Valorization of Fly Ashes and Sands Wastes from Biomass Boilers in One-Part Geopolymers

**DOI:** 10.3390/molecules27206881

**Published:** 2022-10-14

**Authors:** Inês Silveirinha Vilarinho, Marinélia Neto Capela, Ana Sofia Pinho, João António Labrincha, Maria Paula Seabra

**Affiliations:** Department of Materials and Ceramic Engineering, CICECO—Aveiro Institute of Materials, Campus Universitário de Santiago, University of Aveiro, 3810-193 Aveiro, Portugal

**Keywords:** alkali-activated materials, sustainable construction materials, biomass combustion residues, fluidized bed combustion, biomass fly ashes, sustainability

## Abstract

Fly ash (FA) and exhausted bed sands (sands wastes) that are generated in biomass burners for energy production are two of the wastes generated in the pulp and paper industry. The worldwide production of FA biomass is estimated at 10 million tons/year and is expected to increase. In this context, the present work aims to develop one-part alkali-activated materials with biomass FA (0–100 wt.% of the binder) and sands wastes (100 wt.% of the aggregate). FA from two different boilers, CA and CT, was characterized and the mortar’s properties, in the fresh and hardened conditions, were evaluated. Overall, the incorporation of FA decreases the compressive strength of the specimens. However, values higher than 30 MPa are reached with 50 wt.% of FA incorporation. For CA and CT, the compressive strength of mortars with 28 days of curing was 59.2 MPa (0 wt.%), 56.9 and 57.0 MPa (25 wt.%), 34.9 and 46.8 MPa (50 wt.%), 20.5 and 13.5 MPa (75 wt.%), and 9.2 and 0.2 MPa (100 wt.%), respectively. The other evaluated characteristics (density, water absorption, leached components and freeze–thaw resistance) showed no significant differences, except for the specimen with 100 wt.% of CA. Therefore, this work proved that one-part geopolymeric materials with up to 90 wt.% of pulp and paper industrial residues (FA and sand) can be produced, thus reducing the carbon footprint associated with the construction sector.

## 1. Introduction

The demand for products from the pulp and paper industry has been increasing over the years. In 2020, the pulp and paper market size was evaluated at EUR 335 billion [1], and its compound annual growth rate is expected to be 4.08% from 2022 to 2026 [2]. However, as with any industrial process, wastes are generated, such as fly ashes (FA) and exhausted bed sands (ES) or sands wastes, and most of them are landfilled. FA and ES are generated during the high temperature combustion of biomass in the fluidized bed boilers and co-generation power plant. The estimated amount of biomass FA produced worldwide is 10 million tons/year [3]. Therefore, its valorization has gained interest.

In 2020, worldwide CO_2_ emissions were approximately 35 billion tons [4,5]. Cement production accounted for 1.6 billion tons, which represents, approximately, 5% of the global amount [4,5]. Therefore, to reach net zero emission by 2050, a decrease is needed [6]. Hence, efforts are being made to improve the sustainability of cement manufacturing and to develop new construction materials with a lower carbon footprint, such as alkali-activated materials. In this context, the incorporation of FA in construction materials has been studied either on cement-based or alkali-activated materials [7].

In cement-based materials, different substitution levels (15 to 80 wt.%) of coal FA have been tested [7]. Several studies revealed that, in the long term, coal FA can improve the mechanical strength and durability of the material because it consumes the Ca(OH)_2_ generated by cement hydration and produces secondary hydrates such as C-S-H [8]. Nonetheless, some problems arise with this incorporation, such as longer setting times and lower strength at early ages. To overcome these problems, alkaline solutions or compounds have been used to improve the reactivity of FA in hybrid alkaline cements [7].

In the literature, investigations with biomass fly ashes are scarce. However, recently, Capela et al. (2021) [9], studied the use of FA biomass as a partial substitute for OPC (0 to 67 wt.%) and studied the influence of pretreatments on the properties of the prepared mortars. The authors demonstrated that the replacement of 17 wt.% of OPC by as received FA does not affect the properties of the specimens, which resisted up to 25 freeze–thaw cycles.

In the preparation of geopolymeric materials, two routes have been studied: the traditional method, where aluminosilicate materials are mixed with an alkaline solution, and the “one-part” or “just add water” method, where the activator is solid and water is used (similar process to the one used to produce cementitious materials). In this context, Saeli et al. (2019) [10] developed, using the traditional method, geopolymers with two wastes from the pulp and paper industry: biomass FA and grits. For the binder, an optimized mixture of 70 wt.% of FA and 30 wt.% of metakaolin was used, while grits were used as aggregates. The authors concluded that most of the formulated materials can be used in construction and masonry applications (compressive strength higher than 10 MPa). Lu et al. (2022) [11] studied the influence of the type and amount of alkali activator on the strength and microstructure of geopolymers prepared with 100 wt.% of coal FA. The authors used sodium tert-butoxide dissolved in water and sodium silicate powder and their amount varied between 5 and 20 wt.% of FA weight. The mixture of both activators improved the materials’ compressive strength at 28 days of curing. The highest value achieved was 30.6 MPa for the formulations with 5 wt.% of sodium tert-butoxide and 10 wt.% of sodium silicate.

Regarding one-part alkali-activated materials, Oderji et al. (2020) [12] studied the effect of calcium rich slag in aggregate-free FA-based geopolymers. The precursors used were F class FA (coal combustion) and ground granulated blast furnace slag (BFS). The authors concluded that the increase in the slag content improved the compressive and flexural strength; however, the workability was reduced and microcracks were observed when more than 15 wt.% of FA was replaced by the slag. Wan-En et al. (2021) [13] also investigated the effect of different solid activators (anhydrous Na_2_SiO_3_ and NaAlO_2_) on FA-based one-part geopolymers. FA from coal combustion was used, which was mainly composed of silica, alumina, iron and calcium, at values of 36.7, 18.7, 17.2 and 19.1 wt.%, respectively. The amount of activator used varied from 10 to 25 wt.% of FA amount and the authors obtained compressive strengths of 83.6 and 45.1 MPa for anhydrous Na_2_SiO_3_ and NaAlO_2_, respectively, at 28 days of curing.

Despite the numerous works developed on coal FA and sands wastes in the preparation of building materials, studies with biomass FA are still scarce. Furthermore, the development of one-part alkali-activated materials is very recent and they can play an important role in the substitution of cement once their associated carbon footprint is lower than that of the cement. Therefore, the aim of the work is, based on a previous work [14] in which sands wastes were used to develop one-part geopolymer mortars, to study the effect of replacing BFS with biomass FA. The developed materials were characterized in terms of mechanical strength at 7 and 28 days of curing, water absorption, density, leaching and freeze–thaw resistance.

## 2. Results and Discussion

### 2.1. Preparation of One-Part Geopolymer Materials

The composition of the reference sample (paste) and the water/binder ratio (W/B) of the prepared one-part geopolymer are displayed in Table 1. Waste-containing formulations are shown in Table 2 and Table 3, for CA and CT, respectively. In the pastes, a maximum 25 wt.% substitution of BFS by FA was defined since this replacement promoted an accentuated decrease in the final setting time of the mixtures, hindering the conformation of the specimens. This constraint was not observed in the mortars, so several substitution levels were tested (0, 25, 50, 75 and 100 wt.%). For each composition, the W/B ratio was adjusted in order to keep the slump value constant, meaning similar workability. In all formulations, the binder/aggregate ratio used was 0.5 in mass. Furthermore, FA and sand waste used were generated in the same boiler, e.g., when CT fly ashes are used only CTS sand was used as aggregate.

The designation of the prepared compositions adopted the following nomenclature

(1) $$MPC\_\frac{W}{B}$$

where:

*M*—Denoted the mortars materials (MCAS or MCTS);

*P*—Precursor: Blast furnace slag (BFS) or fly ash (CA or CT) used;

*C*—Substitution of BFS by CA or CT in wt.%;

$\frac{W}{B}$—Water/binder ratio.

**Figure 1:**
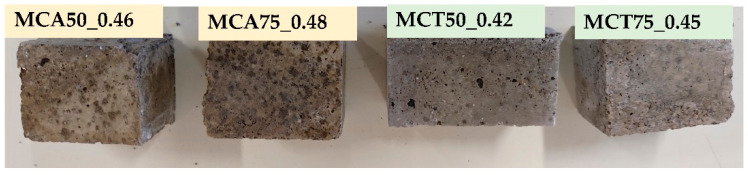
Specimens cured for 28 days: MCT75_0.45, MCT50_0.42, MCA75_0.48 and MCA50_0.46.

The first preparation step was the homogenization of all solid materials (BFS, FA, SM and sand) in a plastic bag. Then, the mixture was mechanically mixed for 60 s while adding distilled water + manual mixing for 30 s + mechanical mixing for 60 s. The prepared mixture was poured into metallic molds (4 × 4 × 4 cm^3^) which were vibrated for 2 min, to remove the trapped air. The molds were placed inside a closed plastic bag for 24 h at ambient temperature. Finally, the specimens were demolded and placed in a climatic chamber at 20 °C and 65% of relative humidity until the 7th and 28th days of curing. Figure 1 shows specimens cured for 28 days.

### 2.2. Raw Materials’ Characterization

The chemical composition of CA and CT fly ashes are shown in Table 4. The main components of CA are sodium oxide (23.3 wt.%), calcium oxide (22.0 wt.%) and chloride (16.6 wt.%), while the main components of CT are silica (39.5 wt.%), calcium oxide (19.6 wt.%) and aluminium oxide (13.1 wt.%). In addition, CA and CT have a loss of ignition (LOI) of 16.10 and 4.41 wt.%, respectively, which might indicate the presence of organic or unburnt matter. The calcium carbonate might also contribute to the LOI value, especially in the case of CA.

Blast furnace slag (BFS), as previously reported by the authors [14], is mainly composed of calcium oxide (46.6 wt.%), silica (32.4 wt.%), aluminum oxide (9.2 wt.%) and magnesium oxide (6.7 wt.%) and has a very low LOI value (0.65 wt.%). The sands wastes are mainly composed of silica and calcium oxide, as reported in a previous work [14].

The geopolymerization involves the chemical reaction between silicates and aluminates in a highly alkaline environment [15]. Thus, the use of CA fly ash, due to its low silica and aluminum oxide content, can negatively influence the reactivity of geopolymeric pastes. However, its relatively high sodium and calcium contents can have the opposite effect.

In cement-based materials, according to EN 197-1:2011, the precursor should have a maximum of 0.1 wt.% chlorides and an LOI ≤ 5 wt.%. For geopolymeric materials there is no standard yet. Nevertheless, since the chloride content in both FA is higher than 0.1 wt.%, leaching tests were performed on the developed materials.

**Figure 2:**
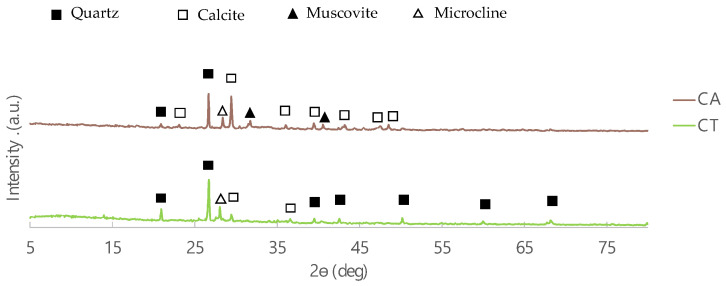
Diffractograms of CA and CT biomass fly ashes.

The diffractograms of the two fly ashes samples (CA and CT) are shown in Figure 2. The main crystalline phases present in both FA are quartz and calcite, which is in agreement with the XRF results (high silica and calcium contents). The highest content of silica in CT is also evident in these diffractograms where the intensity of the quartz peaks are higher. In addition, microcline is present in both fly ashes, but muscovite is not evident in CT.

**Figure 3:**
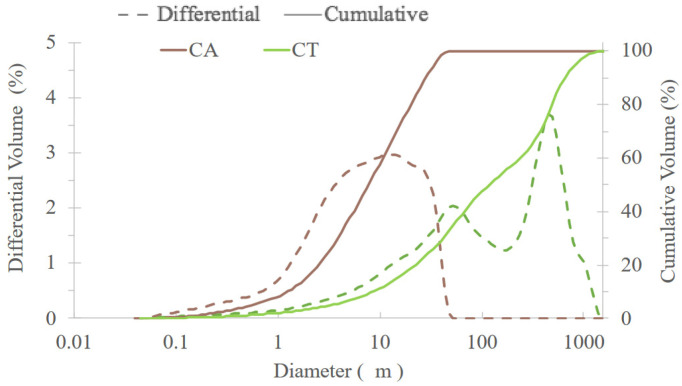
Granulometric distribution of CA and CT biomass fly ashes.

The particle size distribution of CA and CT fly ashes is presented in Figure 3. CA ashes have a much smaller particle size than CT. The D50 values are 8 and 118 μm, respectively, for CA and CT. The maximum particle size is 58 μm for CA and 1660 μm for CT. The particle size of the precursor is very important as it determines the reactivity and, hence, the speed and extent of the geopolymerization reaction. BFS has a D50 of 8.39 μm [14], which is very close to that of CA.

**Figure 4:**
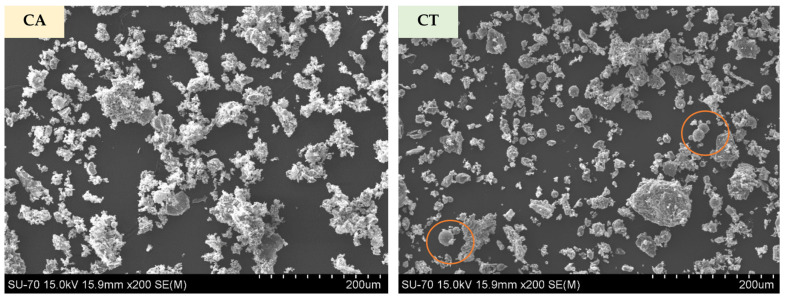
Micrographs of CA and CT biomass fly ashes.

The morphology of both FA particles is presented in Figure 4. The images confirm the higher fineness and the presence of some agglomerates in CA. CT shows a broader range size. Shapes in both cases are irregular; however, some rounded particles are observed in the CT micrograph (examples are highlighted by the orange circles).

### 2.3. Geopolymers’ Characterization

#### 2.3.1. Fresh State

**Figure 5:**
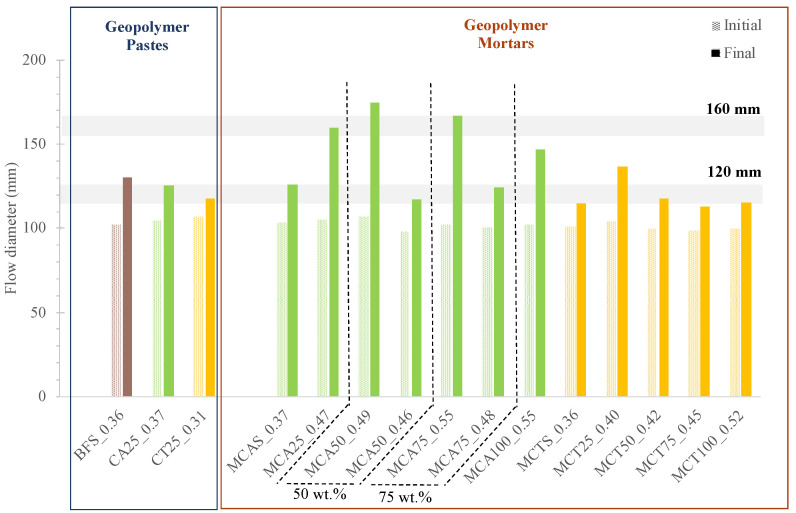
Flow table results of all prepared geopolymer formulations.

The values obtained on the spread table for all the prepared formulations are presented in Figure 5. For geopolymeric pastes, the targeted final flow diameter was 120 mm. A slight increase in water content (W/B of 0.36 vs. 0.37) is required when 25 wt.% of BSF is replaced by CA. The formulation containing CT is more fluid and the required W/B ratio is lower (0.31). This behaviour is related with the larger particle size of this waste. On the contrary, CA ashes have an average particle size close to BFS, so their replacement does not significantly change the amount of water required to maintain the fluidity.

In mortars, increasing the FA content, regardless of the fly ash type, leads to an increase in the need of water to achieve the same final flow diameter. For example, W/B ratios of 0.36, 0.42 and 0.52 for 0, 50 and 100 wt.% CT, respectively.

**Figure 6:**
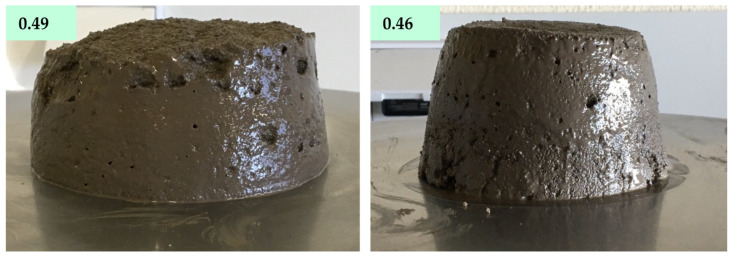
Appearance of MCA50 prepared with a W/B ratio of 0.49 and 0.46 after the flow table test.

On MCA50 mortar the decrease in W/B, from 0.49 to 0.46 W/B, caused a decrease in the final flow diameter, from 175 to 117 mm. This change is easily denoted by the appearance of the materials after the flow table test, see Figure 6.

#### 2.3.2. Hardened State

**Figure 7:**
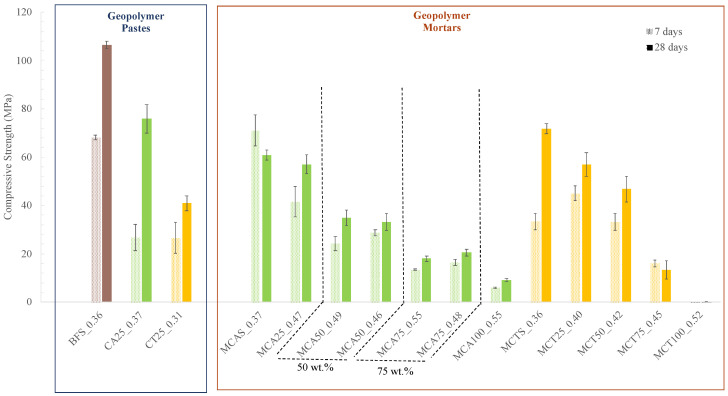
Compressive strength of the hardened samples after 7 and 28 days of curing.

The compressive strength of the hardened samples (pastes and mortars) with 7 and 28 days of curing is presented in Figure 7. In the case of geopolymeric pastes, it was observed that, for both curing times, the compressive strength of the BSF0.36 sample is higher than that of the samples prepared with 25 wt.% of FA (CA25_0.37 and CT25_0.31). The decrease is more evident in the formulation with CT, which, after 28 days of curing, exhibits a compressive strength of 41 MPa. For the same curing time, the reference (BSF_0.36) exhibits a compressive strength of 106 MPa and the sample with CA (CA25_0.37) 76 MPa. This behaviour may be due to the particle size distribution and the lower reactivity of both fly ashes. Nevertheless, all the values of the compressive strength are still very high (>40 MPa).

All prepared mortars showed a lower mechanical strength than the respective pastes. This was expected since the aggregates are not reactive and assume the major fraction in the mixture. A decrease in compressive strength is observed with the increasing of the FA content, which is more pronounced for the samples prepared with the CA. However, the mechanical strength required for mortars depends on their application. Hence, considering only this characteristic, all mortars with up to 50 wt.% of CT (46.8 MPa) and up to 75 wt.% of CA (20.5 MPa) can be used for foundations and retaining walls where a compressive strength greater than 18 MPa is required [16]. Samples with 75 wt.% of CT (13.5 MPa) can be used in coatings and floors where the required compressive strength is 13 MPa. Samples without BSF (100 wt.% of CA) exhibit a compressive strength of 9.2 MPa, which makes them suitable to be used in walls as the minimum required is 5 MPa [16].

Furthermore, as expected, a higher W/B ratio leads to a slight decrease in the strength of the specimens, i.e., MCA75_0.55 and MCA75_0.48 show 17.9 and 20.5 MPa, respectively, at the 28th day of curing. In this case, the water is in excess and is not used in the geopolymerization reaction; therefore, this water evaporates, originating porosity in the material and decreasing its mechanical strength. No differences were observed in the geometric density of the specimens with 7 and 28 days of curing. The obtained values vary between 1.8 and 2.2 g/cm^3^.

The results of the water absorption by immersion are displayed in Table 5. In geopolymeric pastes an increase in water absorption is observed with the use of FA, which is more evident when CT is used, 0.53, 0.8 and 2.5% for BSF, CA and CT, respectively. Regarding the geopolymeric mortars, the highest values were obtained for MCA100_0.55 and MCT75_0.45, 2.7 and 3.7%, respectively. CA fly ash has smaller particles than CT, so it can act as filler; consequently, mortars are more compact and the water absorption is lower. The specimen with 100 wt.% CT disintegrated in water and, therefore, it was impossible to calculate its water absorption by immersion. This is a clear indication of its inadaptability for use in exterior applications, despite the measured mechanical strength. In addition, as expected, the increase in the W/B ratio leads to a rise in the water absorption, 0.65 and 1.0% for MCA50_0.46 and MCA50_0.49, respectively. In this case, the water is in excess and, when the curing evaporates, this creates porosity in the mortars, allowing, therefore, the absorption of more water.

**Figure 8:**
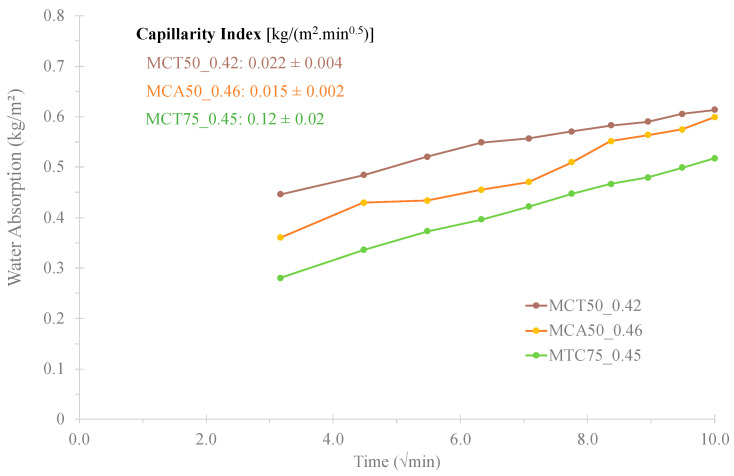
Water absorption by capillarity of MCT0.42FA50, MCA0.46FA50 and MCT0.45FA75 specimens.

The results of the water absorption by capillarity and the capillarity index of the most promising formulations (MCT50_0.42, MCA50_0.46 and MCT75_0.45) are displayed in Figure 8. All values are lower than the ones of OPC mortars (0.5–1.2 kg/(m^2^·min^0.5^)) [17], 0.022 ± 0.004, 0.015 ± 0.002 and 0.12 ± 0.02 kg/(m^2^ min^0.5^) for MCT50_0.42, MCA50_0.46 and MCT75_0.45, respectively. Among all tested specimens, the one that presents the lowest capillarity index is the MCA50_0.46, which means that is the formulation with the lowest interconnected porosity. These results are consistent with the previous data of water absorption by immersion: 0.65, 1.62 and 3.68% for MCA50_0.46, MCT50_0.42 and MCT75_0.45, respectively.

**Figure 9:**
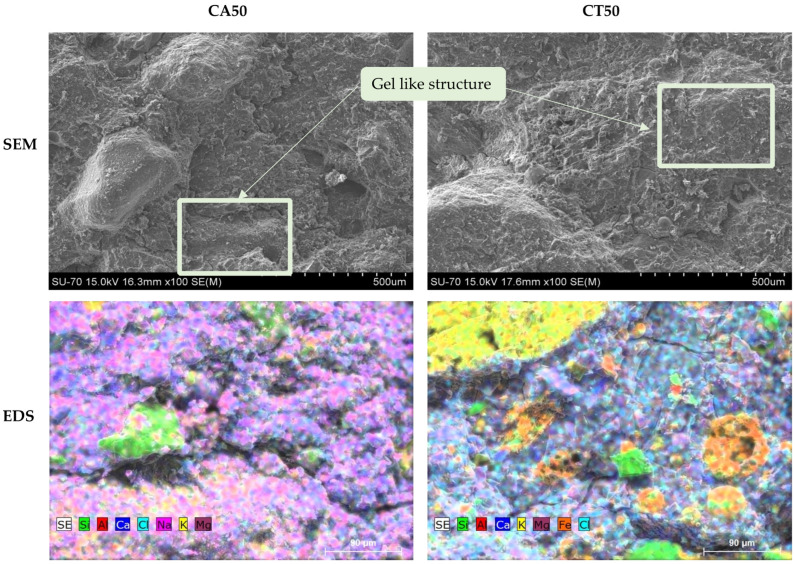
SEM and EDS micrographs of the fracture surface of the MCT50_0.42 and MCA50_0.46 specimens.

SEM and EDS micrographs of the geopolymeric mortars with 50 wt.% of FA are presented in Figure 9. Both FA seem well incorporated in the geopolymeric matrix and the geopolymerization occurred due to the gel-like appearance, as identified inside the rectangular shape. Furthermore, looking at the EDS micrographs in the mortar with CA, the components seem better dispersed than in CT. In the mortar with CT, there are clusters of potassium and iron certainly associated to the coarser particles. Moreover, it can be depicted that the rounded particles of CT are mainly composed of iron.

**Figure 10:**
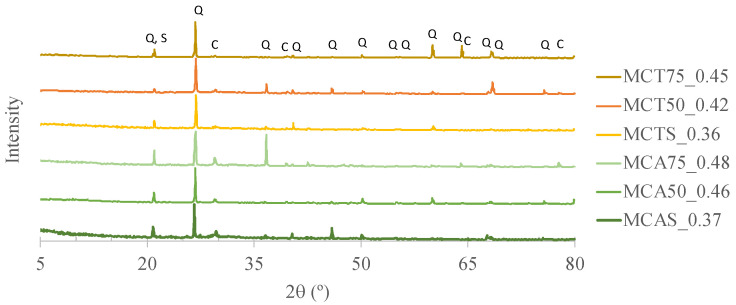
X-ray diffraction (XRD) patterns of specimens after 28 days of curing where Q stands for quartz, C for calcite and S for sodium aluminosilicate.

Figure 10 presents the XRD patterns of the specimens after 28 days of curing. The main crystalline phases are quartz, calcite and sodium aluminosilicate (probably microcline that was already present in the raw materials). These results are in agreement with the XRF results of the sand and biomass fly ash (see Table 4).

Leaching tests were performed on specimens cured at 28 days to evaluate the mobility of selected species. For this, the specimens were submerged in distilled water with a liquid/solid ratio of 10 L/kg with agitation during 24 ± 0.5 (according to EN 12457-2 [18]). In addition, the specimens were kept submerged in distilled water for a total of 48 ± 0.5 h to guarantee that no further leaching occurred.

The most relevant leached concentration results, after specimens’ submersion for 48 ± 0.5 h in distilled water, are displayed in Table 6. Other elements, such as vanadium, arsenic and rubidium were also analysed, but for all of them, the concentrations in the leachate were lower than 0.1 ppm. Observing Table 6, it can be noticed that all the leachate values are below 70 ppm and, therefore, it can be concluded that the components are well imbibed/immobilized in the geopolymer matrix. Moreover, no differences between the leached concentrations at 24 and 48 h were observed.

The mortars formulated with CTS sand (MCTS_0.36) present very low leached chloride amounts, 1.10 ± 0.01 ppm. However, when CAS sand is used, this amount increases to 3.30 ± 0.02 ppm, which is due to the larger abundance of this species in the waste [14].

Regarding the mortars prepared with FA, the highest leached amount of sodium (Na) was obtained for the samples with CA (<50 ppm). The specimens with CT exhibited a lower value (<30 ppm). These results are in agreement with XRF results since CA is much richer in sodium than CT, 23 and 1.1 wt.%, respectively. The leached amount of sulfur increases with FA content; nonetheless, the values are still very low, <11 and <6 ppm for the samples prepared with CA and CT, respectively. The same behaviour is observed for the leached content of chloride (Cl) and potassium (K). These results are in agreement with those obtained in the XRF of the fly ashes since CA contains a higher quantity of Cl and K than CT, 16.6 and 7.7 wt.% for CA and 1.33 and 6.49 wt.% for CT, respectively. The leached calcium amount increases with the use of FA; however, all values are below 1 ppm.

### 2.4. Freeze–Thaw Tests

**Figure 11:**
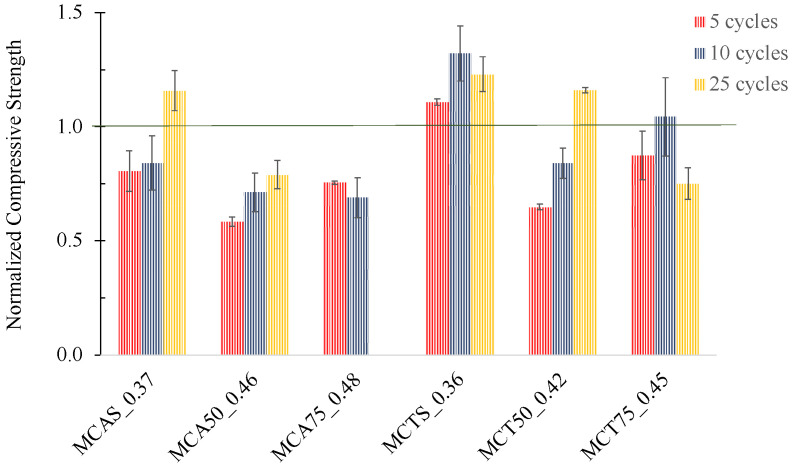
Normalized compressive strength of specimens with 5, 10 and 25 cycles of freeze–thaw tests.

The results of MCAS_0.37, MCTS_0.36, MCA50_0.46, MCA75_0.48, MCT50_0.42 and MCT75_0.45 specimens in the freeze–thaw cycles are shown in Figure 11. Normalization was performed taking into account the corresponding strength at 28 days of curing, so prior to the freeze–thaw testing.

The physical integrity of all the specimens was kept in all cases, excepting the MCA75_0.48 sample that started to degrade after the 10th cycle. Mortars with CTS sand showed an increase in the compressive strength by 20 ± 7 wt.%. The mortars with CAS sand presented a decrease in the mechanical strength for 5 and 10 cycles but an increase of 15 ± 9 wt.% was observed in the samples after 25 cycles. This increase might be explained by the heating step (60 °C) of the cycles that can promote the incomplete geopolymerization reactions.

Regarding the mortars with FA, after 25 freeze–thaw cycles, CT shows a better behaviour, compared with CA, since the compressive strength increases with 50 wt.% of FA. Furthermore, specimens with 75 wt.% of CT were also able to support all the 25 cycles, while CA did not. Nevertheless, due to the high compressive strength of the developed materials (see Figure 8), the samples with 50 wt.% of both FA (CA and CT) and with 75 wt.% of CT can withstand extreme conditions up to 75 days.

## 3. Materials and Methods

### 3.1. Materials

The solid precursors used were blast furnace slag (BFS), from Ecocem (France), and two fly ashes (FA), CA and CT, from the fluidized bed biomass boilers of The Navigator Company, Portugal, that were used in the as received condition. Granular sodium metasilicate powder (SM, Na_2_O = 47–49.5 wt.%, SiO_2_ = 50.5–53 wt.%, 122.06 g/mol), from Sigma-Aldrich (USA), was used as alkaline activator.

For mortars preparation, two sands wastes (CAS and CTS) were collected from the same boilers as the FA (from The Navigator Company, Portugal). The particle size of the commercial sand is between 62 μm and 2 mm, therefore the sands were sieved to adapt their particle size distribution. Furthermore, in all prepared samples (pastes and mortars), distilled water was used.

### 3.2. Raw Materials’ Characterization

X-ray fluorescence (XRF, Phillips X’Pert PRO MPD spectrometer, Amsterdam, The Netherlands) was used to determine the chemical composition and the loss on ignition (LOI) at 1000 °C of the FA. The particle size distribution of the FA was obtained by laser diffraction in a Coulter LS analyzer (LS 230 model, CA, USA). X-ray diffraction (XRD, Rigaku Geigerflex D/max-Series instrument, Tokyo, Japan) was used to evaluate the mineralogical composition of the samples and the phase identification was performed using the PANalytical X’Pert HighScore Plus PRO3 software (Almelo, The Netherlands). Furthermore, scanning electron microscopy (SEM, Hitachi S4100, Tokyo, Japan, 25 kV acceleration voltage) was used to evaluate the microstructure of the fly ashes. All this characterization was also performed in the sands wastes and is present in our previous work [14].

### 3.3. Geopolymer Characterization Tests

In the fresh state, the workability of all the prepared compositions was evaluated by the flow table test, according to EN 1015-3 [19].

The hardened state properties of the samples were evaluated after 28 days of curing (20 °C and 65% RH). The compressive strength of the samples cured for 7 days was also measured. The following properties were assessed:

(i) Compressive strength using a Universal Testing Machine (AG-25TA Shimadzu, Kyoto, Japan) with a displacement rate of 0.5 mm/min, according to EN 1015-11 [20];
(ii) Geometric density, determined from the weight and geometric volume;
(iii) Water absorption (WA) by immersion, 24 h in water, AW(%) = (m_w_ − m_d_)/m_d_ × 100 where m_w_ is the wet mass and m_d_ the dry mass;
(iv) Capillary water absorption, according to EN 1015-18 [21]; herein, the specimens were dried and immersed in 5 to 10 mm of water height. The samples’ weight was measured over time until a maximum time of 90 min;
(v) Microstructure and semi-quantitative elemental composition by scanning electron microscopy (SEM, Hitachi S4100, 15 kV acceleration voltage) equipped with an energy dispersion spectroscopy system (EDS) (Bruker, QUANTAX 400);
(vi) X-ray diffraction (XRD) of milled specimens (PANalytical XPert PRO diffractometer, Ni-filtered CuKa radiation, PIXcel 1D detector, and the exposition corresponded to about 2 s per step of 0.02° 2θ at room temperature).
(vii) Leaching behaviour of the monolithic materials according to EN 12457-2, after 24 and 48 h [18]. The amount of leached components was evaluated in a total reflection X-ray fluorescence spectrometer (TXRF-S2 PICOFOX 50 keV), a more detailed description is presented in [22].

Three replicates were used in each test and the respective mean and associated standard deviation error were calculated.

### 3.4. Durability Tests

The specimens MCA_0.37, MCT_0.36, MCA50_0.46, MCA75_0.48, MCT50_0.42 and MCT75_0.45 were subjected to durability tests (freeze–thaw resistance). For this purpose, the specimens were dried in an oven at 60 °C for 24 h, immersed in water at room temperature for 24 h and then frozen at −18 °C for 24 h—which corresponds to 1 cycle. This procedure was repeated 5, 10 and 25 times, which corresponds to a total of 75 days. In the end, the compressive strength was evaluated. Three replicates were used and the respective average and standard deviation error values were calculated.

## 4. Conclusions

Biomass fly ashes (FA)-based one-part alkali-activated materials were developed in the present work. The precursor, a blast slag furnace, was substituted with two biomass FA: CA and CT. CA exhibits a lower particle size (12 μm) than CT (255 μm) but is closer to the reference material (8 μm). Furthermore, CA has a lower silica and alumina content than CT ash, a higher loss on ignition (16.10% CA and 4.41% CT) and a higher chloride content (16.55% CA and 1. 33% CT).

The results obtained, in the fresh and hardened states, for the geopolymeric pastes and mortars allow these conclusions to be made:

- Specimens formulated with CA exhibit a slightly higher mechanical strength than those prepared with CT. This behaviour should be related to the particle size of the FA and, consequently, to its reactivity.
- The compressive strength of the samples decreases with increasing FA incorporation and, consequently, the possible amount of FA incorporation depends on the intended application.
- Leaching of the FA components was below 70 ppm.
- Developed compositions support 25 freeze–thaw cycles, except the one with 75 wt.% of CA.

Therefore, this work proves that it is possible to develop one-part alkali-activated materials with pulp and paper industry residues. Furthermore, considering the use of 75 wt.% of FA, up to 90 wt.% of residues (fly ash and sand) can be used. This value-added application will not only impact the pulp and paper industry but will also reduce the carbon footprint associated with the construction sector.

## Figures and Tables

**Table 1:** Compositions of the prepared reference samples (paste).

Paste	**Reference**	**BFS (g)**	**SM (g)**	**W Sand (g)**	**W/B Ratio**
	BFS_0.36	100	10	-	0.36

**Table 2:** Compositions of the prepared materials with residues from the CA boiler.

Paste	**Reference**	**BFS (g)**	**CA** **Fly Ash (g)**	**SM (g)**	**CAS** **Sand (g)**	**W/B Ratio**
	CA25_0.37	75	25	10	-	0.37
Mortars	MCAS_0.37	100	-		200	0.37
	MCA25_0.47	75	25			0.47
	MCA50_0.49	50	50			0.49
	MCA50_0.46	50	50			0.46
	MCA75_0.55	25	75			0.55
	MCA75_0.48	25	75			0.48
	MCA100_0.55	-	100			0.55

**Table 3:** Compositions of the prepared materials with residues from the CT boiler.

Paste	**Reference**	**BSF (g)**	**CT** **Fly Ash (g)**	**SM (g)**	**CTS** **Sand (g)**	**W/B** **Ratio**
	CT25_0.31	75	25	10	-	0.31
Mortars	MCTS_0.36	100	-		200	0.36
	MCT25_0.40	75	25			0.40
	MCT50_0.42	50	50			0.42
	MCT75_0.45	25	75			0.45
	MCT100_0.52	-	100			0.52

**Table 4:** Chemical composition and loss on ignition of CA and CT biomass fly ashes.

Component	Na_2_O	MgO	Al_2_O_3_	SiO_2_	P_2_O_5_	SO_3_	Cl	K_2_O	CaO	Fe_2_O_3_	LOI
CA (wt.%)	23.33	1.64	1.62	5.25	0.71	2.99	16.55	7.67	22.00	1.28	16.1
CT (wt.%)	1.13	2.76	13.13	39.47	1.20	2.29	1.33	6.49	19.62	6.44	4.41

**Table 5:** Water absorption by immersion of the specimens with 28 days of curing.

	Specimens	Water Absorption (%)
Geopolymer Pastes	BFS_0.36	0.5 ± 0.1
	CA25_0.37	0.9 ± 0.1
	CT25_0.31	2.5 ± 0.4
Geopolymer Mortars	MCAS_0.37	0.5 ± 0.1
	MCA25_0.47	0.6 ± 0.1
	MCA50_0.49	1.0 ± 0.1
	MCA50_0.46	0.6 ± 0.1
	MCA75_0.55	1.1 ± 0.5
	MCA75_0.48	0.9 ± 0.1
	MCA100_0.55	2.7 ± 0.1
	MCTS_0.36	2.0 ± 0.3
	MCT25_0.40	1.1 ± 0.5
	MCT50_0.42	1.7 ± 0.1
	MCT75_0.45	3.8 ± 0.1
	MCT100_0.52	-

**Table 6:** Leached concentration results after specimens’ submersion for 48 ± 0.5 h in distilled water.

Specimens	Components (ppm)				
	Na	S	Cl	K	Ca
Distilled water	<25	-	<0.5	<0.5	<1
MCAS_0.37	<25	2.34 ± 0.02	3.30 ± 0.02	8.29 ± 0.02	0.48 ± 0.03
MCA50_0.46	<50	10.8 ± 0.1	41.2 ± 0.1	68.9 ± 0.1	0.9 ± 0.01
MCA75_0.48		3.6 ± 0.1	11.5 ± 0.1	15.7 ± 0.1	0.58 ± 0.03
MCTS_0.36	<30	1.57 ± 0.03	1.10 ± 0.01	3.74 ± 0.01	0.48 ± 0.01
MCT50_0.42		4.31 ± 0.03	9.10 ± 0.03	14.45 ± 0.03	0.86 ± 0.01
MCT75_0.45		5.3 ± 0.1	16.2 ± 0.1	25.2 ± 0.1	0.89 ± 0.01

## Data Availability

Not applicable.

## Sample Availability

Samples of the compounds are not available from the authors.
